# Phylogeography of the Rickett’s big-footed bat, *Myotis pilosus* (Chiroptera: Vespertilionidae): a novel pattern of genetic structure of bats in China

**DOI:** 10.1186/1471-2148-13-241

**Published:** 2013-11-05

**Authors:** Guanjun Lu, Aiqing Lin, Jinhong Luo, Dimitri V Blondel, Kelly A Meiklejohn, Keping Sun, Jiang Feng

**Affiliations:** 1 Jilin Key Laboratory of Animal Resource Conservation and Utilization, Northeast Normal University, Changchun 130117, China; 2 Key Laboratory for Wetland Ecology and Vegetation Restoration of National Environmental Protection, Northeast Normal University, Changchun 130117, China; 3 Department of Evolutionary Anthropology, Duke University, Durham, NC 90383, USA; 4 Department of Biology, University of Florida, Gainesville, Florida 32611, USA

**Keywords:** Genetic structure, Demographic history, Multiple refugia, Male-mediated dispersal

## Abstract

**Background:**

China is characterized by complex topographic structure and dramatic palaeoclimatic changes, making species biogeography studies particularly interesting. Previous researchers have also demonstrated multiple species experienced complex population histories, meanwhile multiple shelters existed in Chinese mainland. Despite this, species phylogeography is still largely unexplored. In the present study, we used a combination of microsatellites and mitochondrial DNA (mtDNA) to investigate the phylogeography of the east Asian fish-eating bat (*Myotis pilosus*).

**Results:**

Phylogenetic analyses showed that *M. pilosus* comprised three main lineages: A, B and C, which corresponded to distinct geographic populations of the Yangtze Plain (YTP), Sichuan Basin (SCB) and North and South of China (NSC), respectively. The most recent common ancestor of *M. pilosus* was dated as 0.25 million years before present (BP). Population expansion events were inferred for populations of Clade C, North China Plain region, Clade B and YunGui Plateau region at 38,700, 15,900, 4,520 and 4,520 years BP, respectively. Conflicting results were obtained from mtDNA and microsatellite analyses; strong population genetic structure was obtained from mtDNA data but not microsatellite data. The microsatellite data indicated that genetic subdivision fits an isolation-by-distance (IBD) model, but the mtDNA data failed to support this model.

**Conclusions:**

Our results suggested that Pleistocene climatic oscillations might have had a profound influence on the demographic history of *M. pilosus*. Spatial genetic structures of maternal lineages that are different from those observed in other sympatric bats species may be as a result of interactions among special population history and local environmental factors. There are at least three possible refugia for *M. pilosus* during glacial episodes. Apparently contradictory genetic structure patterns of mtDNA and microsatellite could be explained by male-mediated gene flow among populations. This study also provides insights on the necessity of conservation of *M. pilosus* populations to conserve this genetic biodiversity, especially in the areas of YTP, SCB and NSC regions.

## Background

Global climatic oscillations have profoundly influenced species’ distributions and their genetic traits [1], and as such, the impact has been the focus of a number of European and North America studies [1,2]. The affects of environmental change associated with Pleistocene climatic oscillations on the evolutionary history and genetic structure of many species in East Asia have also been documented [3,4]. It is important to highlight that the extensive glacial advance that occurred in Europe and North America during the Pleistocene did not occur in China, as numerous monsoons throughout the period acted as a preventative [5]. Consequently, China was never completely covered by continental ice during the multiple episodes of glaciation in the last 2.6 million years [6]. The complicated topographical condition and the change of local/regional climate in China was mostly a direct result of the Tibetan Plateau uplift, rather than extensive glaciation [7]. Thus, most of China had a relatively mild climate during the Pleistocene [8].

In the context of complex regional conditions in China, studies on animal geographic distribution patterns have provided information on the evolutionary histories following the global Pleistocene climate changes [3,4]. Using such studies it is possible to compare the affects of climate changes during the Pleistocene on evolutionary history to regions of similar latitude [9]. Previous studies suggested that colonization events, dispersal patterns and migratory behaviors play a crucial role in determining a species’ geographical range and demographic history [3,4,10]. In addition, both historical events and ecological factors also influence extant species genetic diversity and population structure [11]. Ice-age refugia, which provide a chance of surviving for species, have been proposed to been present in the Southeast China Hills region, Sichuan Basin region and Yunnan region, however this still remains controversial. Some studies have shown that cold condition forced species below 25° N in eastern China [12]. Others have argued that a decrease in temperature would have reduced the overall species range, but that a range of relatively stable mountain microclimates would have allowed species to persist over much of their range [13,14]. Research on the phylogeography of Chinese flora and fauna during the Pleistocene has been limited, with many species remaining unexplored including non-sedentary bats.

Bats are the second largest order of mammals, and are important biological indicators as they are sensitive to both climate and environmental changes [15]. Previous studies have revealed that the demographic history and current geographical distribution of many bats were influenced by ancient climatic changes [16,17]. In mainland China, population genetic structure can often be explained by the influence of local geographical structure. All previous studies on Chinese bats have shown that subpopulations originally existed in eastern China [11,18,19]. However, with differences among geographical structure, multiple subpopulations in the mountains of southern China region (Yungui Plateau region, Sichuan Basin region and Southeast China Hills region) have also been documented [11,19]. For example, two subpopulations existed in this region for *Myotis davidii*; one in the Yungui Plateau region and the other in the Southeast China Hills region [18]. A similar trend was noted for *Hipposideros armiger* by Lin and colleagues [11], who noted four subpopulations of *H. armiger* in this region. These studies have illustrated that the population genetic structure of bats is consistent with this region’s geographical structure. As the dispersal of most bat species is male-biased, examining the maternally inherited mitochondrial DNA (mtDNA) along with microsatellites can provide insights into genetic population structure. [16,17,20-23].

**Figure 1:**
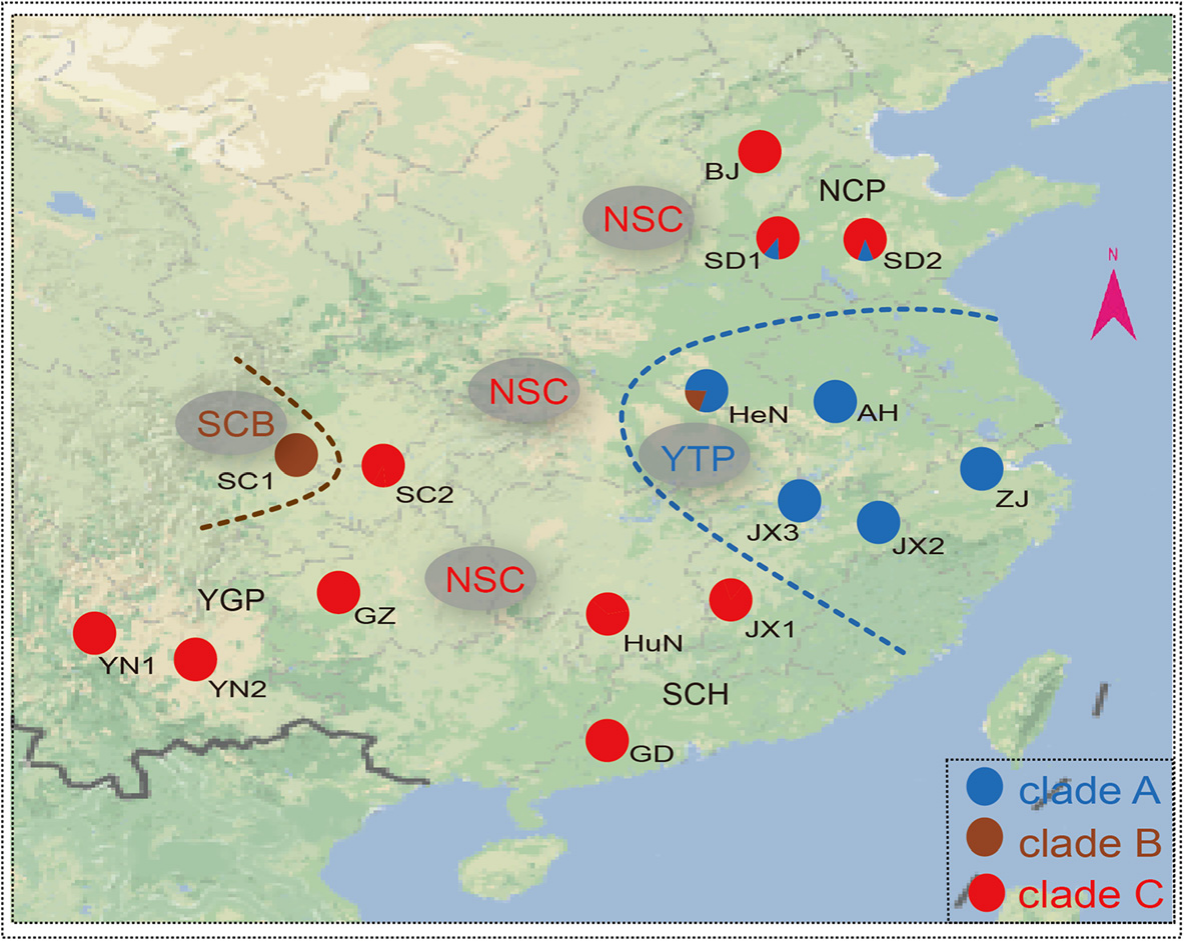
**Map of China showing 16 sampling populations.** The color pie charts refer to 3 halpotype lineages in Bayesian phylogenetic tree. The abbreviations of sampling populations are consistent with those in Table 1.

There are three dominant fish-eating bat species found globally: the greater bulldog bat (*Noctilio leporinus*) and the Mexican fishing bat (*Myotis vivesi*), mostly found in the New World tropics, as well as the Rickett’s big-footed bat (*Myotis pilosus*), exclusively distributed in the Old World temperate regions [24]. In China, *M. pilosus* is abundant and mainly distributed in North China Plain (NCP) region, Southeast China Hills (SCH) region, YunGui Plateau (YGP) region, Sichuan Basin (SCB) region and Yangtze Plain (YTP) region (five geographical units; Figure 1). A single fish species, *Zacco platypus*, makes up 60% of the diet for *M. pilosus*[25]. Importantly, *M. pilosus* has been listed as a “Near Threatened” species by The International Union for Conservation of Nature (IUCN) [26].

Considering that bats are good model organisms for assessing environmental and climatic changes, and that during the Pleistocene China did not experience extreme glaciation, we explored the phylogeography of *M. pilosus* using both mtDNA and microsatellite markers to address some questions: 1) Were population divergence and expansion events of *M. pilosus* related to Pleistocene climate change? 2) Did glacial refugia exist in Eastern China during the Pleistocene? 3) Were the spatial distribution of mtDNA lineages of *M. pilosus* consistent with the geographical structure*?* and 4) Did mtDNA and microsatellite markers provide the same genetic structure patterns for *M. pilosus*?

## Results

### Sequence characteristics and genetic diversity

**Figure 2:**
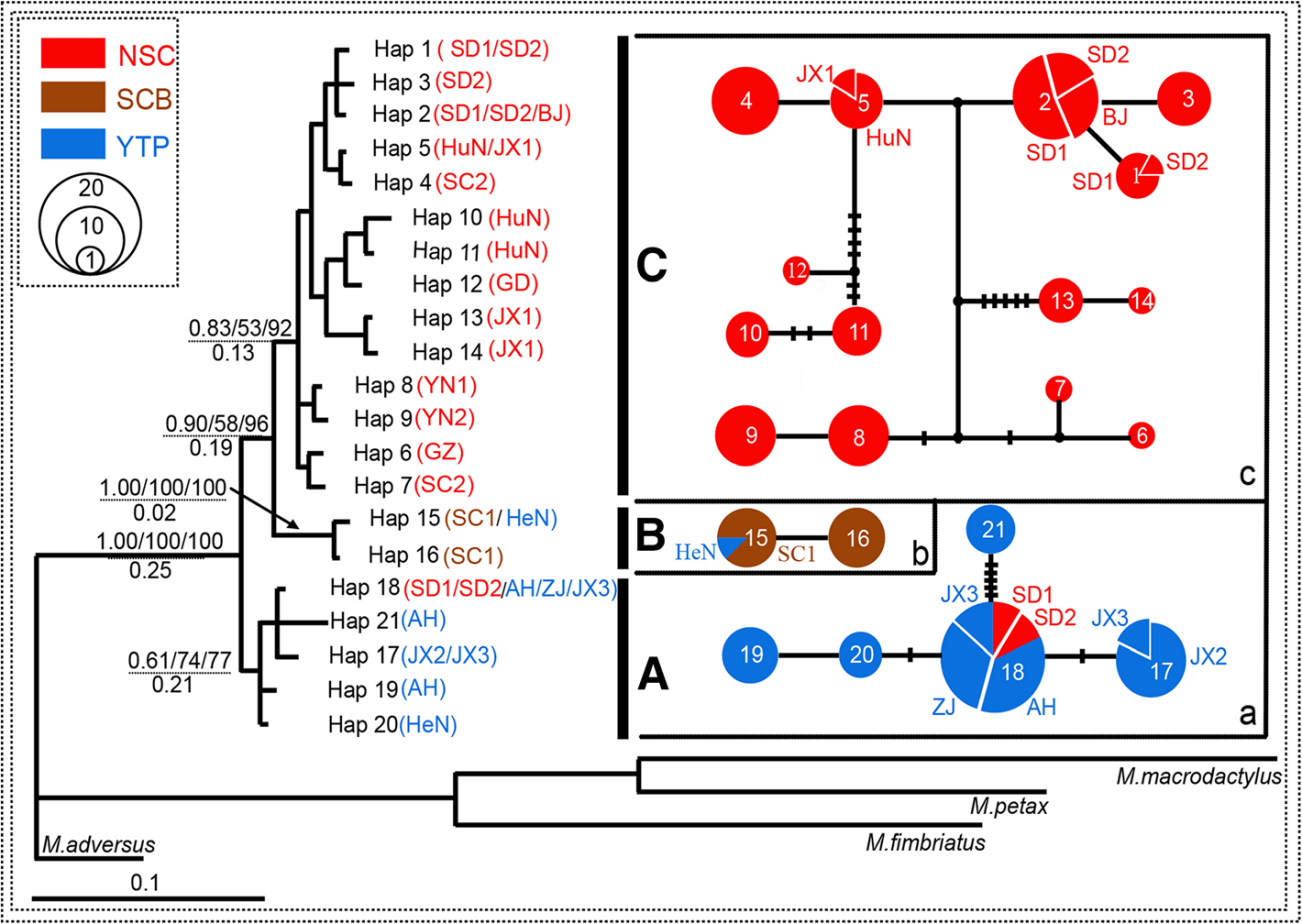
**Bayesian (BI) phylogenetic tree and TCS networks for *Myotis pilosus *based on mtDNA.** Neighbour-joining (NJ), maximum likelihood (ML) and Bayesian (BI) methods resulted in concordant topologies with three major lineages (Clade A, B and C). Bootstrap values (BI/ML/NJ, above the line) above 50% and time of the most recent common ancestor (million years ago, below the line) were shown on major lineage nodes. The black dots represented missing steps between observed haplotypes. The short vertical bars referred to substitutions between haplotypes. Circle sizes were proportional to haplotype frequency (denoted in left upper corner). The haplotype numbers in networks were consistent with those in BI phylogenetic tree.

The mitochondrial control region varied in length (685 to 847 bp) as a result of a 81 bp repeat (which was present a minimum of four times). For consistency, only the first repeat (Additional file 1, sites from 267 to 347) was included in subsequent analyses, given a total length of 442 bp. In total, we identified 21 haplotypes from the 146 sequences. Forty-three polymorphic sites and three insertions or deletions (Additional file 1, sites 45, 342 and 350) were observed. Fifteen haplotypes of these (71.43%) were unique within a single population. Four haplotypes (Hap 1, 2, 5, 17) were shared by populations within the same geographical unit. Two haplotypes were shared by different geographical units: Hap 15 was shared by SC1 from SCB region and HeN from YTP region; and Hap 18 was shared by NCP region (SD1 and SD2) and YTP region (ZJ, AH and JX3) (Figure 1 and Figure 2). All haplotypes were submitted to GenBank (Additional file 2).

The overall haplotype diversity (*h*) was 0.932, and the overall nucleotide diversity (π) was 0.022. The microsatellite analysis showed expected heterozygosities (*He*) ranged from 0.68 for SC1 to 0.84 for JX3, and observed heterozygosity (*Ho*) ranged from 0.57 for SC1 to 0.89 for YN1. Allelic diversity (*Rs*) was 4.56 (Table 1). No consistent departure from HWE or signs of linkage disequilibrium was detected (all *P* > 0.05), and the mean frequencies of null alleles for each locus were low (all *r* < 0.05). The analysis showed that our dataset had high power (> 90%) of detecting population structure even at a low level of genetic differentiation (*F*st = 0.005, Additional file 3), and α error was less to 5% in all cases.

**Table 1:** **Genetic variability of *Myotis pilosus *** **based on mtDNA and microsatellite data**

**Population**	**N**	**mtDNA**			**Microsatellite**		
		**H**	***h***	***π***	***He***	***Ho***	***Rs***
Jinan, Shandong (SD1)	17	3	0.588 ± 0.093	0.008 ± 0.004	0.76 ± 0.14	0.76 ± 0.16	4.16
Linyi, Shandong (SD2)	16	4	0.542 ± 0.103	0.008 ± 0.003	0.73 ± 0.21	0.61 ± 0.11	3.99
Fangshan, Beijing (BJ)	5	1	-	-	0.68 ± 0.19	0.60 ± 0.32	3.81
**North China Plain (NCP)**	**38**	**4**	**0.676 ± 0.055**	**0.007 ± 0.002**	-	-	-
Ji’an, Jiangxi (JX1)	6	3	0.500 ± 0.215	0.006 ± 0.004	0.76 ± 0.13	0.58 ± 0.38	4.31
Yongzhou,Hunan (HuN)	14	3	0.714 ± 0.052	0.011 ± 0.001	0.77 ± 0.13	0.77 ± 0.32	4.23
Shaoguan, Guangdong (GD)	1	1	-	-	-	-	-
**Southeast China Hills (SCH)**	**21**	**6**	**0.824 ± 0.040**	**0.014 ± 0.001**	-	-	-
Xuanhan, Sichuan (SC2)	10	2	0.200 ± 0.154	0.003 ± 0.002	0.71 ± 0.24	0.66 ± 0.29	4.07
Baoshan, Yunnan (YN1)	8	1	-	-	0.82 ± 0.09	0.89 ± 0.08	4.57
Kunming, Yunnan (YN2)	8	1	-	-	0.79 ± 0.08	0.70 ± 0.26	4.32
Anlong, Guizhou (GZ)	1	1	-	-	-	-	-
**YunGui Plateau (YGP)**	**27**	**5**	**0.738 ± 0.037**	**0.008 ± 0.001**	-	-	-
Beichuan, Sichuan (SC1)	14	2	0.538 ± 0.052	0.001 ± 0.0001	0.68 ± 0.35	0.57 ± 0.35	4.12
**SiChuan Basin (SCB)**	**14**	**2**	**0.538 ± 0.052**	**0.001 ± 0.0001**	-	-	-
Xinyang, Henan (HeN)	5	2	0.400 ± 0.237	0.004 ± 0.008	0.76 ± 0.29	0.65 ± 0.40	4.89
Liushun, Anhui (AH)	20	3	0.589 ± 0.043	0.011 ± 0.007	0.73 ± 0.22	0.75 ± 0.30	4.33
Jinhua, Zhejiang (ZJ)	7	1	-	-	0.73 ± 0.23	0.68 ± 0.24	4.66
Guangfeng, Jiangxi (JX2)	9	1	-	-	0.74 ± 0.20	0.61 ± 0.13	4.34
Wuyuan, Jiangxi (JX3)	5	2	0.600 ± 0.175	0.003 ± 0.001	0.84 ± 0.11	0.84 ± 0.22	4.72
**Yangtze Plain (YTP)**	**46**	**6**	**0.763 ± 0.038**	**0.008 ± 0.001**	-	-	-
**Total**	**146**	**21**	**0.932 ± 0.008**	**0.022 ± 0.001**	-	-	-

The number of individuals sampled (N), number of haplotypes observed (H), mean and standard deviation of haplotype diversity (*h*), nucleotide diversity (*π*), expected heterozygosities (*He*), observed heterozygosities (*H*o) and allelic richness (*Rs*) for the studied *Myotis pilosus*.

### Phylogenetics

Neighbor-joining (NJ), maximum likelihood (ML), and Bayesian (BI) analysis recovered identical topologies. The phylogenetic trees recovered monophyly of the whole *M. pilosus* population and assigned all the haplotypes into three main phylogenetic lineages: Clade A (YTP lineage), Clade B (SCB lineage) and Clade C (NSC lineage) (Figure 1 and Figure 2). Clade A included the most individuals from YTP region (Figure 1) and four individuals from SD1 and SD2. Clade B was restricted to all individuals from SC1, but there was however one individual from HeN. Clade C was comprised of mostly specimens from YGP region, SCH region and NCP region (Figure 1). The TCS analysis of the mtDNA data produced three independent networks (networks a, b and c) (Figure 2).

For mtDNA results, AMOVA revealed significant genetic variance for all three hierarchical levels examined (among regions, among populations within regions, and within populations, all *P* < 0.001), and strong population structure with 69.56% of the genetic variation found among regions. The microsatellite data however showed weak population structure, with 95.41% of the variation coming from within a population (Table 2).

**Table 2:** **Analysis of molecular variance (AMOVA) based on mtDNA and microsatellite of *Myotis pilosus *** **with three geographical regions: Yangtze plain (YTP) region, Sichuan basin (SCB) region and North and South of China (NSC) region**

	**d.f.**	**Sum of squares**	**Variation (%)**	**Fixation indices**
**mtDNA**				
Among regions	2	472.62	69.56	Φ_CT_ = 0.70***
Among populations within regions	11	179.36	19.89	Φ_SC_ = 0.65***
Within populations	130	102.16	10.57	Φ_ST_ = 0.83***
**Microsatellites**				
Among regions	2	8.22	0.11	Φ_CT_ = 0.01**
Among populations within regions	9	44.92	4.60	Φ_SC_ = 0.05***
Within populations	278	498.84	95.41	Φ_ST_ = 0.05***

Statistically significant results were indicated by asterisks: **P < 0.01, ***P < 0.001.

**Figure 3:**
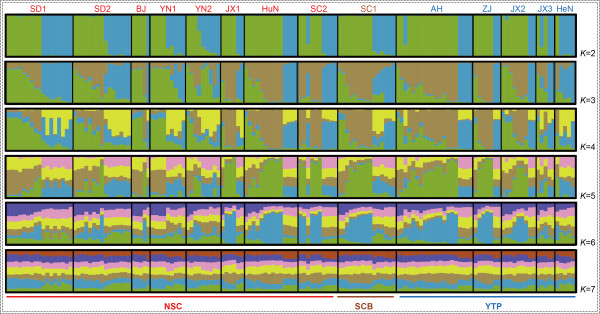
**Bayesian clustering results (*K*** **= 2–7) performed in STRUCTURE for *Myotis pilosus*.** The abbreviations of sampling localities were consistent with those in Table 1.

The STRUCTURE result at *K* = 2 provided the best separation and Delta *K* value (= 374). However, individuals from the same population were recovered with multiple groups based on microsatellite data, indicating a mixed population structure. Similar results were also found when *K* = 3–6. Most of the individuals from YN1, YN2, BJ, SD1 and SD2 were assigned to a cluster, and individuals from JX1, HuN, SC1, SC2, AH, ZJ and JX2 were assigned to a separate cluster. No marked groups were identified when *K* > 6 (Figure 3).

Individual assignment tests indicated that only a small proportion of individuals of *M. pilosus* (25%) were assigned to the populations from which they were sampled; instead most individuals (75%) were assigned to other populations. Among the three genetic lineages identified from the mtDNA data, 30 (62.5%) of the individuals from Clade A, 13 (81.25%) from clade B, 42 (51.13%) from clade C, were assigned to other genetic lineages.

**Figure 4:**
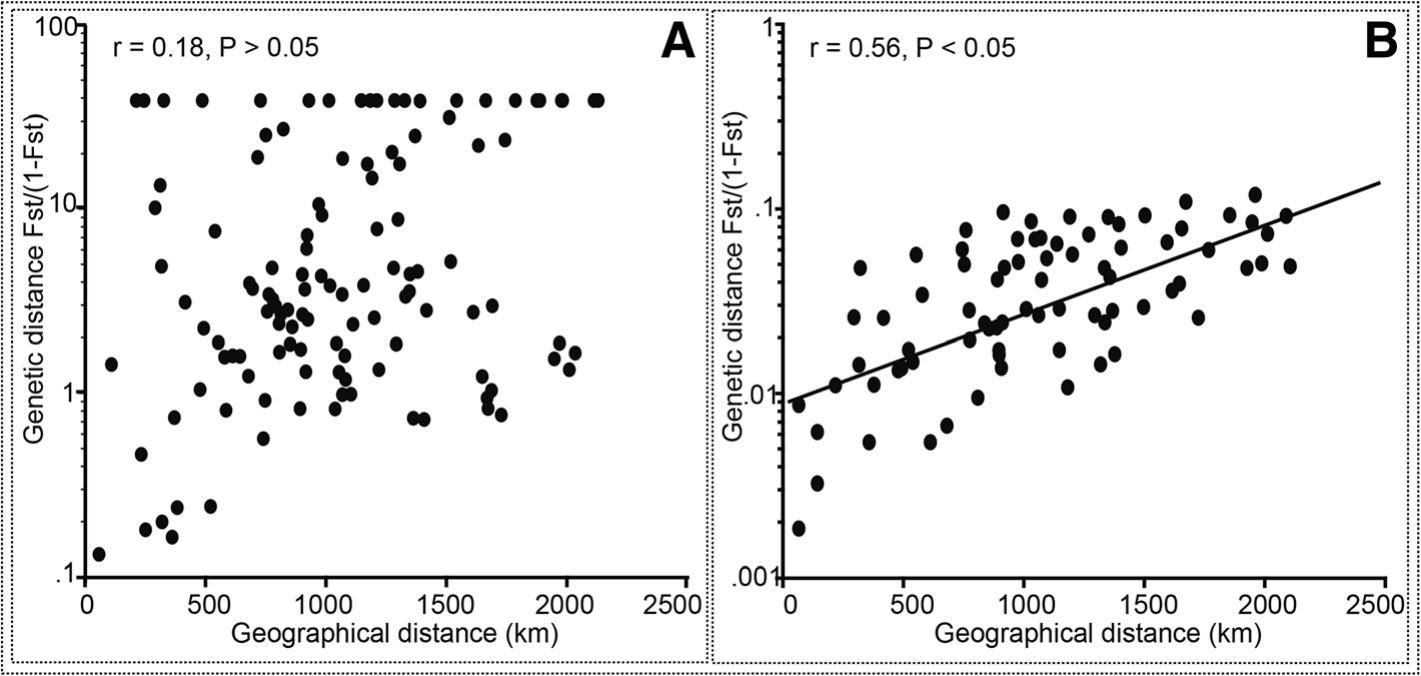
Plot of genetic distance based on mtDNA (A) and microsatellite data (B) versus geographical distance for pairwise population comparisons.

The overall *F*st value of mtDNA was 0.83 (*P* < 0.001). Only 5.83% of the pairwise *F*st values were not significant (*P* > 0.05) in the whole population, suggesting the existence of significant population differentiation in *M. pilosus* (Additional file 4a). For microsatellite analysis, the overall *F*st value was 0.05 (*P* > 0.05). Up to 54.95% of pairwise *F*st were not significant (*P* > 0.05) in the whole population, and Nm values suggested more gene flow in microsatellite data than in mtDNA (Additional file 4). A Mantel test indicated that mtDNA failed to support isolation-by-distance (IBD) model (*r* = 0.21, *P* > 0.05; Figure 4A), however, microsatellite marker fitted an IBD model (*r* = 0.56, *P* < 0.001; Figure 4B).

### Demographic history

BEAST inferred the time of the most recent common ancestor (TMRCA) for all sequences as 0.25 (95% CI 0.12-0.40) million years (Myr) BP. Similar TMRCA estimates obtained for Clade B/C were 0.19 (95% CI 0.09-0.31) Myr BP. The TMRCA for Clade A, B and C, were 0.21 (95% CI 0.13-0.29), 0.02 (95% CI 0.01-0.05) and 0.13 (95% CI 0.06-0.21) Myr BP, respectively (Table 3).

**Table 3:** **Results of mismatch distribution analyses and neutrality tests for *Myotis pilosus *** **sampled in YunGui plateau (YGP, including population YN1, YN2, GZ, and SC2) region, Southeast China hills (SCH, including JX1, HuN and GD) region, and North China plain (NCP, including BJ, SD1and SD2) region, clade A (A), Clade B (B), Clade C (C)**

**Clade**	**Neutrality test**			**Mismatch distribution**				**Time since expansion (95% CI) (years)**
	**Tajima’s D**	**Fu’s Fs**	***R***_**2**_	**SSD**	**Rag**	**Md**	**tau (95% CI)**	
**A**	0.32	3.87	0.72	0.66*	0.34	Multimodal	0	-
**B**	1.5	1.31	0.32*	0.03	0.28	Unimodal	0.80 (0.30–3.45)	4,520 (1,270–19,500)
**C**	−0.18	0.53	0.10*	0.01	0.01	Unimodal	7.20 (2.15–11.15)	38,700 (12,100–63,100)
**YGP**	−0.7	0.61	0.12*	0.04	0.18	Unimodal	0.80 (0.50–1.70)	4,520 (2,610–9,100)
**SCH**	1.22	3.42	0.65	0.09*	0.17	Multimodal	0.77 (0.45–1.26)	-
**NCP**	1.25	2.06	0.13*	0.01	0.04	Unimodal	3.00 (1.56–5.69)	15,900 (8,840–32,100)

Statistically significant results were indicated by asterisks: * *P* < 0.05.

All tests showed the expansion events for Clade B, Clade C, and expansion times were 4,520 years BP (95% CI 1,270-19,500), and 38,700 years BP (95% CI 12,100-63,100), respectively. In more detail, within Clade C, NCP (BJ, SD1 and SD2) region and YGP (YN1, YN2, GZ and SC2) region were found the expansion events for 15,900 years BP (95% CI 8,840-32,100) and 4,520 years BP (95% CI 2,610-9,100) (Table 3 and Additional file 5).

## Discussion

### Demographic history and expansion events

The glacial refugium hypothesis and the expansion-contraction model predict that long-term isolation of populations within geographically separate refugia will lead to spatially distinct genetic lineages during the Pleistocene [27]. In our study, the results of phylogenetic and median-joining network analyses identified three deeply split lineages of *M. pilosus* in mainland China, arisen due to the presence of multiple glacial refugia during the Pleistocene. The most recent common ancestor of these *M. pilosus* populations is likely to have originated in the Yangtze plains region, as high genetic diversity and ancient haplotypes, and TMRCA could date back to 0.25 Myr BP (Figure 2). At this time, mainland China had just concluded the second stage of penultimate glaciation (0.28-0.27 Myr BP). Thus the Yangtze plains region was likely to be a glacial refuge, supporting our hypothesis that such refugia existed in eastern China during the Pleistocene. Other studies have also confirmed that mountainous areas of this region had relatively stable climatic conditions during the Pleistocene glaciation, providing probable glacial refugia for many species [14,28]. Our study identifies that intra-specific divergence within *M. pilosus* first occurred in 0.25-0.19 Myr BP (Clade A), which corresponds to a relatively warm interglacial period in mainland China [29]. It is possible that the intra-specific divergence noted among *M. pilosus* during this time is a direct result of geographic isolation, resulting from spread of individuals from the YTP region to other regions. The second divergence, Clade B and Clade C in Figure 2, is dated at 0.19-0.13 Myr BP, and is likely associated with increased cold conditions during the third phase of the penultimate glaciation (0.15-0.14 Myr BP) [29]. It is possible that many individuals were able to survive these cold conditions in different but isolated shelters found in distinct refugia. According to our mtDNA results, refugia for Clade C may be located in SCH region, which had the highest haplotype and nucleotide diversity (Table 1). This region has previously been identified as a refuge during the penultimate glaciation, for organisms including *M. davidii*[18], *Rhinolophus ferrumequinum*[28]. Unfortunately, it is not possible to infer the refuge location for Clade B. Overall, our results indicate that population divergences of *M. pilosus* were related to the Pleistocene climatic changes.

In addition to mediating the population divergence of *M. pilosus*, we hypothesized that the Pleistocene climate changes also affected population expansion events. The population expansion tests conducted indicated that the population expansion for Clade C occurred around 38,700 years BP, which correlates to one of the warmest periods during the last glaciation [30]. From our results we were also able to infer that other rapid expansion events occurred from 15,900 to 4,520 years BP for SCB region, NCP region and YGP region (Table 3). This time frame is associated with the end of the last glacial age, where climatic conditions were suitable for population growth. Expansion during this time was plausible and has been documented for a range of species [5,31,32]. These results indicate that the current populations of *M. pilosus* for those regions were derived recently, providing an explanation as to the low genetic diversity in the three geographic units [33]. We also detected several overlap points among divergent mtDNA lineages. For example, the HeN population comprises haplotypes of Clade A and B, and SD1 and SD2 population includes haplotypes of Clade A and C. This overlap could have occurred as a result of a second contact caused by recent population dispersal, or incomplete lineage sorting. It is worth mentioning that although the mtDNA revealed the population history of *M. pilosus*, it does have its limitations [34,35]. Therefore, we expect that when mtDNA is combined with nuclear DNA data in future studies, a more well supported and informative demographic history will be obtained.

### Population structure

As previously mentioned, the mtDNA data identified that individuals from all populations were grouped into three distinct genetic lineages (Figure 1 and Figure 2): Yangtze Plain lineage (Clade A), Sichuan Basin lineage (Clade B) and North and South of China lineage (Clade C: including NCP region, YGP region and SCH region). These population genetic structures were also supported by the results of an AMOVA with 69.56% variation among regions (Table 2). Population genetic structure was generally consistent with the known macro-ecological characteristics, with individuals from populations with similar vegetation types, temperature, precipitation and topography recovered in the same lineage [7,18]. The pattern of subdivision into subpopulations could be explained by the influence of environmental characteristics during an extended period of time, and especially be reflected in mtDNA. We observed that different lineages were located in different geographic regions, such as YTP region for Clade A and SCB region for Clade B. Consistency between population genetic structure and environmental characteristics also existed for other bats species across this same region, such as *M. davidii*[18] and *R. ferrumequinum*[28]. However, the population genetic structure of *M. pilosus* was incomplete consistent with other bats. Interestingly, other studies of Chinese bats which focused on populations from the southern mountains of China (SCH region and YGP region), revealed 2–4 distinct lineages [4,11,18]. In this study we only identified a single genetic lineage of *M. pilosus* (NSC lineage), which included individuals from the NCP region and the southern mountains of China region (SCH region and YGP region) (Figure 1 and Figure 2). This genetic structure difference between *M. pilosus* and other bats species might be explained by differences in the number of refugia. In this geographic region, *M. pilosus* survived in a single glacial shelter (such as the SCH region), while other bats species likely survived in multiple refugia, allowing for a greater number of genetic lineages [18].

The results of genetic differentiation (AMOVA) revealed strong population genetic structure in the mtDNA data; however, similar analyses based on microsatellite showed weak population genetic structure (Figure 3 and Table 2). The different levels of genetic structure in mtDNA and microsatellite may be explained in part by the different divergent mutation rates and/or the effective population size of the maternally and bi-parentally inherited markers [36]. However, we hypothesized that different level of genetic structure between these two data types may be largely ascribed to sex-biased gene flow. There were three possible explanations that led us to this conclusion. Firstly, we observed contrasting patterns of genetic structure among the two markers. Both phylogenetic trees and median-joining network of mtDNA grouped a number of populations, including JX1, HuN, SD1, SD2, YN1, YN2 and SC2 grouped into Clade C, while Clade B only comprised of a single population (SC1; Figure 2). However, the structure analysis based on microsatellite showed that *M. pilosus* individuals classified three distinct lineages, Clade A (ZJ, AH, JX2, JX3 and HeN), Clade B (SC1), and Clade C (JX1, HuN) by mtDNA, but were recovered as a single cluster using microsatellite data (Figure 3). Secondly, only the microsatellite data fit the IBD model from the Mantel test (Figure 4). It is possible that the mtDNA did not support this IBD model as gene flow even among populations, even those separated by short geographic distances, may have been limited [37]. For example, population JX1 is geographically close to JX2 (Figure 1), however, based on mtDNA, they belong to two distinct lineages (recovered in the phylogenetic trees and the median-joining network analysis) (Figure 2). Thus, in spite of having no geographical boundaries to prevent gene flow, it did not occur. This phenomenon also observed among other populations, such as JX1 vs JX2, HuN vs JX2, HuN vs JX3, SC1 vs SC2 (Figure 1). Contrastingly, the analysis of IBD based on the microsatellite data showed a positive relationship between genetic differentiation and geographical distance, indicating gene flow between nuclear lineages. Gene flow among populations using the microsatellite data was supported by the low overall pairwise *F*st, mostly non-significant pairwise genetic differences among populations along with a high Nm (Additional file 4b). The lack of IBD with mtDNA was probably due to female philopatry, as the gene flow between populations was likely mediated by males. Finally, individual assignment tests also indicated frequent migrations among three mtDNA lineages. Combining these results, this study has revealed different genetic variation patterns between mtDNA and microsatellite markers, which may have resulted from the male-biased dispersal. This dispersal pattern has also been documented in other bat species, e.g., *Eptesicus fuscus*[17], *M. bechsteinii*[38], *M. bechsteinii*[39], *M. myotis*[16], *M. punicus*[40], *Nyctalus noctula*[41], *R. monoceros*[20], *R. ferrumequinum*[9]. We inferred that in breeding avoidance is likely to be the crucial factor driving male dispersal in this species [38].

### Conservation implications

*Myotis pilosus* is the only of the three major fish-eating bats that has an Asian or Old World distribution. Due to the specialized diet of *M. pilosus,* it may be highly susceptible to the lack of food resources common when habitats are destroyed [26]. Genetically divergent populations and their surrounding environments have been recognized in the literature as conservation priorites [42]. Based on this and the mtDNA results obtained in this study, YTP, SCB and NSC regions represent areas of conservation importance (Figure 1). For these regions, we suggest that appropriate measures be taken to preserve their genetic uniqueness. Moreover, based on genetic data, the priority for conservation should be given to SCH region with highest genetic diversity and overlap zones (SD1, SD2 and HeN) with multiple lineages. Protection of both regions populations will protect the majority of diversity and lineages of *M. pilosus.*

## Conclusions

Our research has indicated that several populations of *M. pilosus*, which are distributed throughout mainland China, are monophyletic and have a single common ancestor (which is dated back to 0.25 Myr BP). Population dispersal, genetic divergence and expansion events of *M. pilosus* in China are likely to be associated with Pleistocene climate fluctuations. Both the Yangtze Plain region and Southeast China Hill region may have provided shelters for the survival of individuals during cold glaciation. The degree of genetic differentiation, based on the analysis of mtDNA and microsatellite of *M. pilosus*, suggested a male-mediated gene flow among populations. Overall conservation attention should be given to the areas highlighted in this study, which corresponds to regions with high genetic diversity and population overlap zones.

## Methods

### Sampling

One hundred and forty-six individuals were collected from 16 populations distributed across five geographic units (only 14 populations for microsatellite data as GZ and GD only including one bat were discarded) (Figure 1 and Table 1): North China Plain (NCP) region, Southeast China Hills (SCH) region, Yungui Plateau (YGP) region, Sichuan Basin (SCB) region, Yangtze Plain (YTP) region, covering most of the distributional range of *M. pilosus* in China. For each individual, a three-mm wing membrane biopsy punch was taken, following the nonlethal method described by Worthington Wilmer and Barratt [43]. All field studies followed the regulations of Wildlife Conservation of the People’s Republic of China (Chairman Decree [2004] No. 24) and were approved by National Animal Research Authority in Northeast Normal University, China (approval number: NENU-20080416).

### Mitochondrial DNA amplification and microsatellite genotyping

Total genomic DNA was isolated using a UNIQ-10 column animal genomic DNA isolation kit (SK1206, Sangon, Shanghai, China). Hypervariable region I (HVI) of mitochondrial control region was amplified by polymerase chain reaction (PCR) using primer pair P-F [44]. Five microsatellite loci (G9, D15, G25, A13 and E24) from *M. myotis*[45] and *M. daubentonii*[22] were selected for genotyping in all 144 individuals. Three primers (G25, D15 and G9) were labeled with HEX, while the other primers (E24 and A13) were labeled with FAM. PCRs conditions were performed as outlined by Wilkinson [44]. DNA sequences were edited by BioEdit 7.0.5.3 and aligned using CLUSTAL-X. Genotyping was performed on an ABI 3730 automated DNA sequencer (Applied Biosystems, Inc.), and genotype data were analyzed by GeneMapper ID v3.2 (Applied Biosystems).

### Genetic diversity

For mtDNA analysis, Genetic diversity was computed with the DnaSP, version 4.0 [46] based on the number of haplotypes (H), haplotype diversity (*h*) and nucleotide diversity (*π*). For microsatellite analysis, expected heterozygosity (*He*), observed heterozygosity (*Ho*), Hardy-Weinberg equilibrium (HWE) and linkage disequilibrium were determined with GENEPOP 4.0 [47], and average allelic richness (*Rs*) with FSTAT version 2.9.3 [48]. Null allele frequencies for each locus were estimated using Micro-Checker[49].

The program POWSIM 4.0 [50] was used to evaluate the statistical power provided by the five polymorphic loci to detect population structure. Both fisher’s exact test and traditional chi-square approaches were used to test the genetic homogeneity of two best groups from STRUCTURE analysis (the number of individuals was 68 and 72 for each group). Ne/t (Effective size of subpopulations/means generations of drift before sampling) combinations respectively corresponded to 500/5, 1,000/10, 2,000/20, 5,000/50 (as *F*st = 0.005). Estimates of the statistical α error were generated using samples drawn directly from the base population, omitting the drift steps (t = 0) leading to the absence of differentiation (*F*st = 0). In all cases, 1,000 replicates were run and the estimate of power was indicated by the proportion of significant outcomes (*P* < 0.05).

### Phylogenetic analysis

Phylogenetic analyses included neighbor-joining (NJ) performed in PAUP 4.0b10 [51], maximum likelihood (ML) in Phyml 2.4.4 [52] and Bayesian inference (BI) in MRBAYES 3.2.1 [53]. The HKY + G (G = 0.36) model was selected as the best corrected Akaike Information Criterion model (AICc) using jModelTest 0.1.1 [54]. Statistical support for branching patterns was estimated by 1,000 bootstrap replications for NJ and ML. In Bayesian analyses, two independent parallel runs of four incrementally heated Metropolis-coupled MCMCs (Monte Carlo Markov Chains) were run with trees sampled every 100 generations for 1,000,000 generations. The analyses were deemed to have converged when the average standard deviation of split frequencies fell below 0.01. The first 10% of the generations were discarded as ‘burn-in’. Trees were rooted with outgroup of *M. adversus*, *M. macrodactylus*, *M. petax* and *M. fimbriatus* (GenBank number see Additional file 2).

An analysis of molecular variance (AMOVA) was performed using Arlequin 3.5 [55] to examine population structure based on mtDNA and microsatellite at three hierarchical levels (among regions, among populations within regions and within populations). Based on the phylogenetic lineages, all populations were grouped into three population genetic structures: Yangtze Plain lineage (including HeN, AH, JZ, JX2 and JX3), Sichuan Basin lineage (including SC1), North and South of China lineage (including SC2, YN1, YN2 and GZ from YGP region; JX1, HuN and GD from SCH region; SD1, SD2 and BJ from NCP region). In addition, a 95% statistical parsimony approach was employed, which was carried out using the program TCS 1.21 [56].

We applied a Bayesian approach with STRUCTURE 2.3 [57] to construct the population structure for microsatellites in which *K* varied from 1 to 14. We performed 10 replicate runs of structure for each *K* value. MCMCs were run for 500,000 cycles with the first 20% cycles discarded as ‘burn-in’ under admixture and independent allele frequencies models. The best *K* value was defined using Delta *K* described by Evanno [58]. Assignment tests were run in Genetic Analysis with Excel 6.0 [59] to identify the origin population.

For mtDNA and microsatellite, pairwise genetic distances (*F*st) were computed with 10,000 permutations in Arlequin 3.5. A Mantel test with 10,000 randomizations was used to assess the correlation between the standardized genetic distance [*F*st/(1-*F*st)] and geographical distances in IBDWS 3.23 [60]. The geographical distance between each pair of sampling localities was calculated by GIS software Arcinfo 9.0 (ESRI Inc.).

### Demographic history

The most recent common ancestor of major evolution lineages were assessed using BEAST 1.6.2 [61] with a strict molecular clock (ucld.stedv = 0.01) and a HKY + G model. In our study, we used a divergence rate of 20% per million years, as this was applied in a study of bats of the genus *Nyctalus*[62], which equates to a mutation rate of 10% subs/site/Ma. The prior parameters were determined in preliminary studies. Finally, we performed three independent runs of 20,000,000 generations, each with a burn-in of first 10% generations, and sampling every 1,000 steps. These three runs were then combined in TRACER 1.5 [63], which was also used to visualize the results of each run, examine the effective sample size (ESS) for each parameter.

Neutrality tests and mismatch distribution analyses can provide hints to infer population demographic events. Tajima’s D, Fu’s Fs and *R*_2_ test were calculated with DnaSP 4.0. Mismatch Distributions were determined according to the Sudden Expansion Model in Arlequin 3.5. We used goodness-of-fit tests based on the sum of squared deviations (SSD) and raggedness index (Rag) to test the significance of fit of distribution. When an expansion model could not be rejected, we estimated the time of expansion (*t*) from *τ* = *2ut*, where τ is calculated as the time to expansion in mutational units, and *u* is the mutation rate per generation for the whole sequence. The *u* is equal to *μgk*, where *μ* is the mutation rate per nucleotide (see above 10%) and *k* is the sequence length. The generation time (*g*) was estimated to be two years [18].

## Competing interests

The authors declare that have no competing interests.

## Authors’ contributions

GL conceived and designed the experiments and carried out the molecular genetic analysis, participated in the manuscript’s design, and drafted the manuscript. JF and KS developed the ideas and obtained funding support. AL participated in the design of the study and performed the statistical analysis. JL helped collect samples. KS, DVB and KAM helped draft the manuscript. All authors read and approved the final manuscript.

## Supplementary Material

Additional file 1 **Variable sites of mtDNA for 21 Haplotypes.** The Arabic numbers at the top of the figure indicate the variable sites. The “.” indicate agreement with haplotype 1 depicted in the first row. The “-” indicate the insertions or deletions. The “︱ → ←︱”indicate the saved first repeat from 267 to 347 with 81 bp. Sites 267 and 347 are not variable sites. Click here for file

Additional file 2 GenBank accession numbers for mtDNA haplotypes and outgroups used in the present study. Click here for file

Additional file 3 **Results of POWSIM simulations assessing the statistical power of microsatellite loci to differentiate populations with *F*st = 0.005.** The results show the proportion of simulations out of 1,000 significant with a critical value of 0.05. Click here for file

Additional file 4 **Population genetic differentiation (*F*st; lower-left) and gene flow (Nm; upper-right) for *Myotis pilosus.*** Additional file 4a for mtDNA and 4b for microsatellite“-” from upper-right indicates the gene flow is infinity. Statistically significant results are indicated by asterisks: * *P* < 0.05. Click here for file

Additional file 5 **Results of mismatch distributions for all populations, three lineages and three geographical units.** The full circles show observed values whereas the open circle represents expected values. Click here for file
