# Supplementary material for: Phylogeography of the Rickett’s big-footed bat, Myotis pilosus (Chiroptera: Vespertilionidae): a novel pattern of genetic structure of bats in China
Source: BMC Evol Biol. 2013 Nov 5;13:241. doi: 10.1186/1471-2148-13-241 (PMC4228257; doi:10.1186/1471-2148-13-241)

**Additional file 1**

**Variable sites of mtDNA for 21 haplotypes.** The Arabic numbers at the top of the figure indicate the variable sites. The “.” indicate agreement with haplotype 1 depicted in the first row. The “-” indicate either an insertion or deletion. The “︱→←︱” indicates the saved first repeat from 267 to 347, which is 81bp. Sites 267 and 347 are not variable sites.


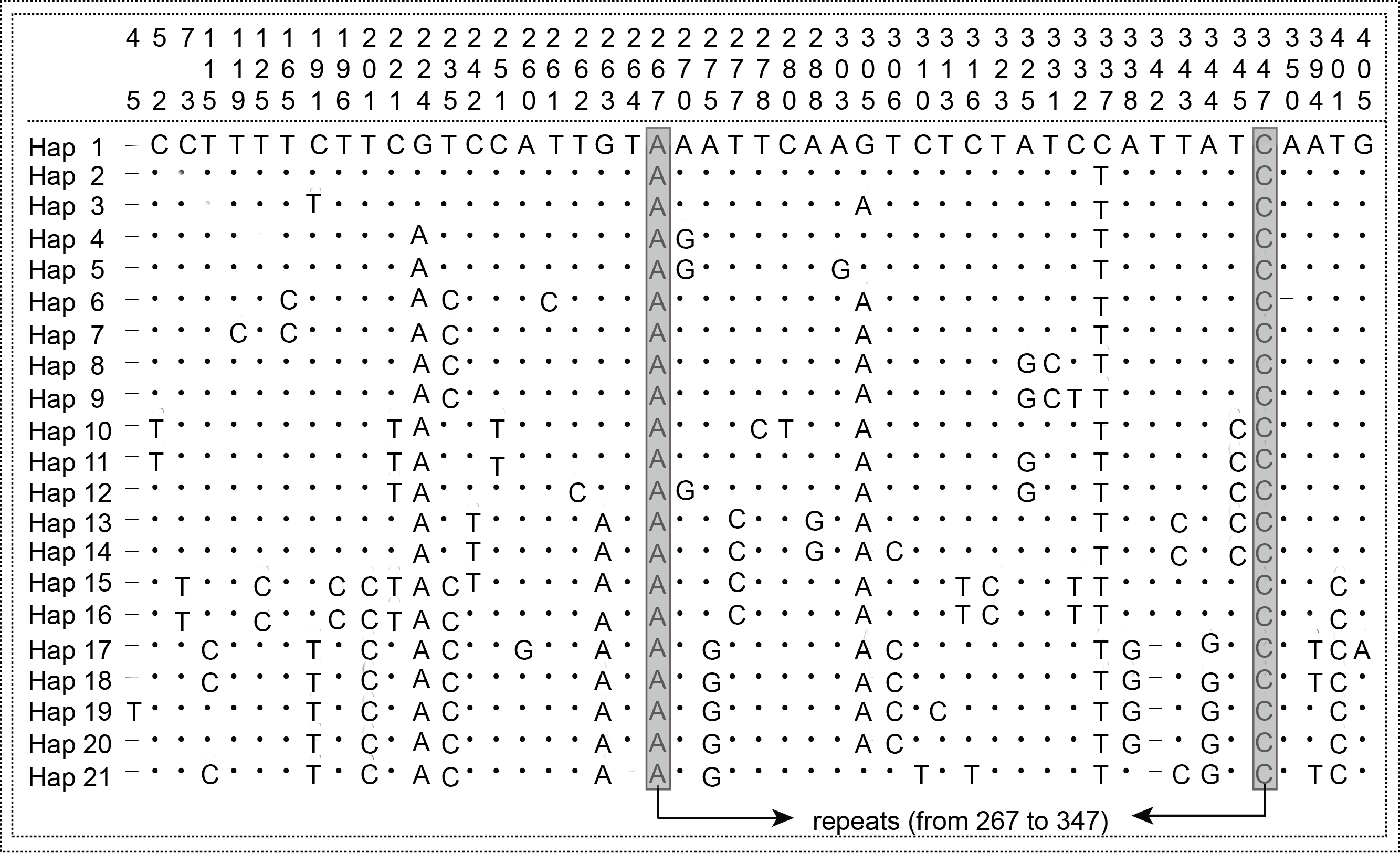

Supplement: Additional file 1 — Variable sites of mtDNA for 21 Haplotypes. The Arabic numbers at the top of the figure indicate the variable sites. The “.” indicate agreement with haplotype 1 depicted in the first row. The “-” indicate the insertions or deletions. The “︱ → ←︱”indicate the saved first repeat from 267 to 347 with 81 bp. Sites 267 and 347 are not variable sites. [file 1471-2148-13-241-S1.doc]
