# Supplementary material for: Phylogeography of the Rickett’s big-footed bat, Myotis pilosus (Chiroptera: Vespertilionidae): a novel pattern of genetic structure of bats in China
Source: BMC Evol Biol. 2013 Nov 5;13:241. doi: 10.1186/1471-2148-13-241 (PMC4228257; doi:10.1186/1471-2148-13-241)
Supplement: Additional file 2 — GenBank accession numbers for mtDNA haplotypes and outgroups used in the present study. [file 1471-2148-13-241-S2.doc]

**Additional file 2**

***GenBank accession numbers for mtDNA haplotypes and outgroups used in the present study.***

| **Haplotype** | **No.** | **Haplotype** | **No.** | **Haplotype** | **No.** | **Outgroup** | **No.** |
| --- | --- | --- | --- | --- | --- | --- | --- |
| Hap1 | KC153407 | Hap8 | KC153414 | Hap15 | KC153421 | *M. adversus* | JF806295 |
| Hap2 | KC153408 | Hap9 | KC153415 | Hap16 | KC153422 | *M. macrodactylus* | JF806310 |
| Hap3 | KC153409 | Hap10 | KC153416 | Hap17 | KC153423 | *M. petax* | JF806312 |
| Hap4 | KC153410 | Hap11 | KC153417 | Hap18 | KC153424 | *M. fimbriatus* | JF806303 |
| Hap5 | KC153411 | Hap12 | KC153418 | Hap19 | KC153425 |  |  |
| Hap6 | KC153412 | Hap13 | KC153419 | Hap20 | KC153426 |  |  |
| Hap7 | KC153413 | Hap14 | KC153420 | Hap21 | KC153427 |  |  |
