# Supplementary material for: Phylogeography of the Rickett’s big-footed bat, Myotis pilosus (Chiroptera: Vespertilionidae): a novel pattern of genetic structure of bats in China
Source: BMC Evol Biol. 2013 Nov 5;13:241. doi: 10.1186/1471-2148-13-241 (PMC4228257; doi:10.1186/1471-2148-13-241)
Supplement: Additional file 3 — Results of POWSIM simulations assessing the statistical power of microsatellite loci to differentiate populations with Fst = 0.005. The results show the proportion of simulations out of 1,000 significant with a critical value of 0.05. [file 1471-2148-13-241-S3.doc]

**Additional file 3 Results of POWSIM simulations assessing the statistical power of microsatellite loci to differentiate populations with *F*st = 0.005**. The results show the proportion of simulations out of 1000 significant with a critical value of 0.05.

| Ne\t  methods | 500\5 | 1000\10 | 2000\20 | 5000\50 |
| --- | --- | --- | --- | --- |
| Chi-squared test | 95.30% | 100% | 100% | 100% |
| Fisher's exact test | 95.56% | 96.50% | 100% | 100% |
