# Supplementary material for: Phylogeography of the Rickett’s big-footed bat, Myotis pilosus (Chiroptera: Vespertilionidae): a novel pattern of genetic structure of bats in China
Source: BMC Evol Biol. 2013 Nov 5;13:241. doi: 10.1186/1471-2148-13-241 (PMC4228257; doi:10.1186/1471-2148-13-241)
Supplement: Additional file 4 — Population genetic differentiation (Fst; lower-left) and gene flow (Nm; upper-right) for Myotis pilosus. Additional file 4a for mtDNA and 4b for microsatellite“-” from upper-right indicates the gene flow is infinity. Statistically significant results are indicated by asterisks: * P < 0.05. [file 1471-2148-13-241-S4.doc]

**Additional file 4a**

**Population genetic differentiation (*F*st; lower-left) and gene flow (Nm; upper-right) estimated using mtDNA data for *Myotis pilosus*.**

|  | SD1 | SD2 | BJ | HeN | AH | ZJ | JX2 | JX3 | JX1 | HuN | GD | SC1 | SC2 | YN1 | YN2 | GZ |
| --- | --- | --- | --- | --- | --- | --- | --- | --- | --- | --- | --- | --- | --- | --- | --- | --- |
| SD1 |  | 3.82 | - | 0.27 | 0.22 | 0.11 | 0.09 | 0.14 | 0.33 | 0.62 | 0.35 | 0.08 | 0.55 | 0.28 | 0.22 | 0.47 |
| SD2 | 0.11 |  | 2.05 | 0.35 | 0.27 | 0.15 | 0.11 | 0.17 | 0.37 | 0.63 | 0.42 | 0.09 | 0.45 | 0.32 | 0.26 | 0.59 |
| BJ | -0.03 | 0.19 |  | 0.15 | 0.16 | 0.00 | 0.00 | 0.02 | 0.14 | 0.54 | 0.00 | 0.01 | 0.16 | 0.00 | 0.00 | 0.00 |
| HeN | 0.64* | 0.58* | 0.76* |  | 2.02 | 0.54 | 0.23 | 0.81 | 0.25 | 0.24 | 0.32 | 0.10 | 0.13 | 0.12 | 0.11 | 0.45 |
| AH | 0.68* | 0.64* | 0.74* | 0.19 |  | 3.04 | 0.61 | 2.75 | 0.18 | 0.18 | 0.17 | 0.10 | 0.14 | 0.15 | 0.13 | 0.23 |
| ZJ | 0.80* | 0.76* | 1.00* | 0.47* | 0.14* |  | 0.00 | 1.00 | 0.05 | 0.10 | 0.00 | 0.01 | 0.02 | 0.00 | 0.00 | 0.00 |
| JX2 | 0.84* | 0.81* | 1.00* | 0.68* | 0.44* | 1.00* |  | 0.30 | 0.03 | 0.08 | 0.00 | 0.01 | 0.01 | 0.00 | 0.00 | 0.00 |
| JX3 | 0.78* | 0.73* | 0.95* | 0.37* | 0.15 | 0.33 | 0.62* |  | 0.08 | 0.12 | 0.03 | 0.02 | 0.04 | 0.01 | 0.01 | 0.04 |
| JX1 | 0.59* | 0.57* | 0.77* | 0.65* | 0.72* | 0.90* | 0.92* | 0.85* |  | 0.42 | 0.27 | 0.05 | 0.15 | 0.08 | 0.07 | 0.23 |
| HuN | 0.44* | 0.44* | 0.47* | 0.66* | 0.72* | 0.82* | 0.85* | 0.79* | 0.54* |  | 2.48 | 0.09 | 0.49 | 0.33 | 0.27 | 0.56 |
| GD | 0.58* | 0.53* | 1.00* | 0.60* | 0.73* | 1.00* | 1.00* | 0.93* | 0.64* | 0.16 |  | 0.01 | 0.12 | 0.00 | 0.00 | 0.00 |
| SC1 | 0.85* | 0.84* | 0.97* | 0.82* | 0.82* | 0.97* | 0.98* | 0.95* | 0.90* | 0.84* | 0.96* |  | 0.02 | 0.01 | 0.01 | 0.01 |
| SC2 | 0.47* | 0.52* | 0.75* | 0.78* | 0.77* | 0.95* | 0.96* | 0.92* | 0.76* | 0.50* | 0.80* | 0.94* |  | 0.06 | 0.05 | 0.11 |
| YN1 | 0.63* | 0.60* | 1.00* | 0.80* | 0.76* | 1.00* | 1.00* | 0.96* | 0.84* | 0.59* | 1.00* | 0.97* | 0.88* |  | 0.00 | 0.00 |
| YN2 | 0.68* | 0.65* | 1.00* | 0.81* | 0.78* | 1.00* | 1.00* | 0.97* | 0.86* | 0.64* | 1.00* | 0.97* | 0.90* | 1.00* |  | 0.00 |
| GZ | 0.51* | 0.45* | 1.00 * | 0.52* | 0.68* | 1.00* | 1.00* | 0.91* | 0.68* | 0.47* | 1.00* | 0.96* | 0.81* | 1.00* | 1.00* |  |

“-” from upper-right indicates the gene flow is infinity. Statistically significant results are indicated by asterisks: * *P* < 0.05.

**Additional file 4b**

**Population** **genetic differentiation (*F*st; lower-left) and gene flow (Nm; upper-right) estimated using microsatellite data for *Myotis pilosus*.**

|  | SD1 | SD2 | BJ | HeN | AH | ZJ | JX2 | JX3 | JX1 | HuN | SC1 | SC2 | YN1 | YN2 |
| --- | --- | --- | --- | --- | --- | --- | --- | --- | --- | --- | --- | --- | --- | --- |
| SD1 |  | 35.82 | 21.47 | 57.60 | 8.63 | 6.13 | 21.89 | 87.55 | 6.45 | 5.30 | 6.34 | 5.43 | 10.55 | 5.69 |
| SD2 | 0.01 |  | 38.56 | - | 13.62 | 9.02 | 24.29 | 34.75 | 9.69 | 5.58 | 16.83 | 8.40 | 9.41 | 6.27 |
| BJ | 0.02 | 0.01 |  | 22.95 | 9.57 | 6.54 | 29.30 | 37.06 | 6.31 | 5.37 | 9.34 | 5.07 | 10.76 | 4.60 |
| HeN | 0.01 | 0.01 | 0.02 |  | 55.70 | 17.04 | - | - | - | 12.47 | 28.47 | 16.16 | - | 10.96 |
| AH | 0.05* | 0.03* | 0.04* | 0.01 |  | - | - | 17.37 | 31.64 | 20.43 | 26.05 | 20.37 | 7.54 | 4.40 |
| ZJ | 0.07* | 0.05* | 0.07 | 0.02 | -0.02 |  | 50.21 | 14.86 | 19.84 | 14.55 | 39.81 | 33.77 | 5.55 | 3.81 |
| JX2 | 0.02 | 0.02 | 0.01 | 0.01 | 0.01 | 0.01 |  | 68.82 | 12.29 | 13.87 | 20.80 | 15.22 | 11.88 | 6.94 |
| JX3 | 0.01 | 0.01 | 0.01 | 0.00 | 0.02 | 0.03 | 0.01 |  | 8.64 | 21.44 | 14.58 | 9.06 | 16.55 | 15.17 |
| JX1 | 0.07* | 0.04* | 0.07 | -0.01 | 0.01 | 0.02 | 0.03 | 0.05* |  | 23.45 | 7.46 | 20.09 | 17.36 | 4.69 |
| HuN | 0.08* | 0.08* | 0.08* | 0.03 | 0.02 | 0.03 | 0.04* | 0.02 | 0.02 |  | 6.48 | 7.48 | 20.48 | 6.40 |
| SC1 | 0.07* | 0.02* | 0.05* | 0.01 | 0.01 | 0.01 | 0.02 | 0.03 | 0.06* | 0.07* |  | 25.23 | 5.03 | 4.81 |
| SC2 | 0.08* | 0.05* | 0.08* | 0.03 | 0.02 | 0.01 | 0.03 | 0.05* | 0.02 | 0.06* | 0.01 |  | 5.27 | 4.61 |
| YN1 | 0.04* | 0.05* | 0.04* | 0.01 | 0.06* | 0.08* | 0.04* | 0.02 | 0.02 | 0.02* | 0.09* | 0.08* |  | 46.87 |
| YN2 | 0.08* | 0.07* | 0.09* | 0.04 | 0.10* | 0.11* | 0.06* | 0.03 | 0.09* | 0.07* | 0.09* | 0.09* | 0.01 |  |

“-” from upper-right indicates the gene flow is infinity. Statistically significant results are indicated by asterisks: * *P* < 0.05.
