# Supplementary material for: Phylogeography of the Rickett’s big-footed bat, Myotis pilosus (Chiroptera: Vespertilionidae): a novel pattern of genetic structure of bats in China
Source: BMC Evol Biol. 2013 Nov 5;13:241. doi: 10.1186/1471-2148-13-241 (PMC4228257; doi:10.1186/1471-2148-13-241)

**Additional file 5**

**Results of mismatch distributions for all populations, three clades and three geographical units.** The full circles show observed values whereas the open circles represent expected values.


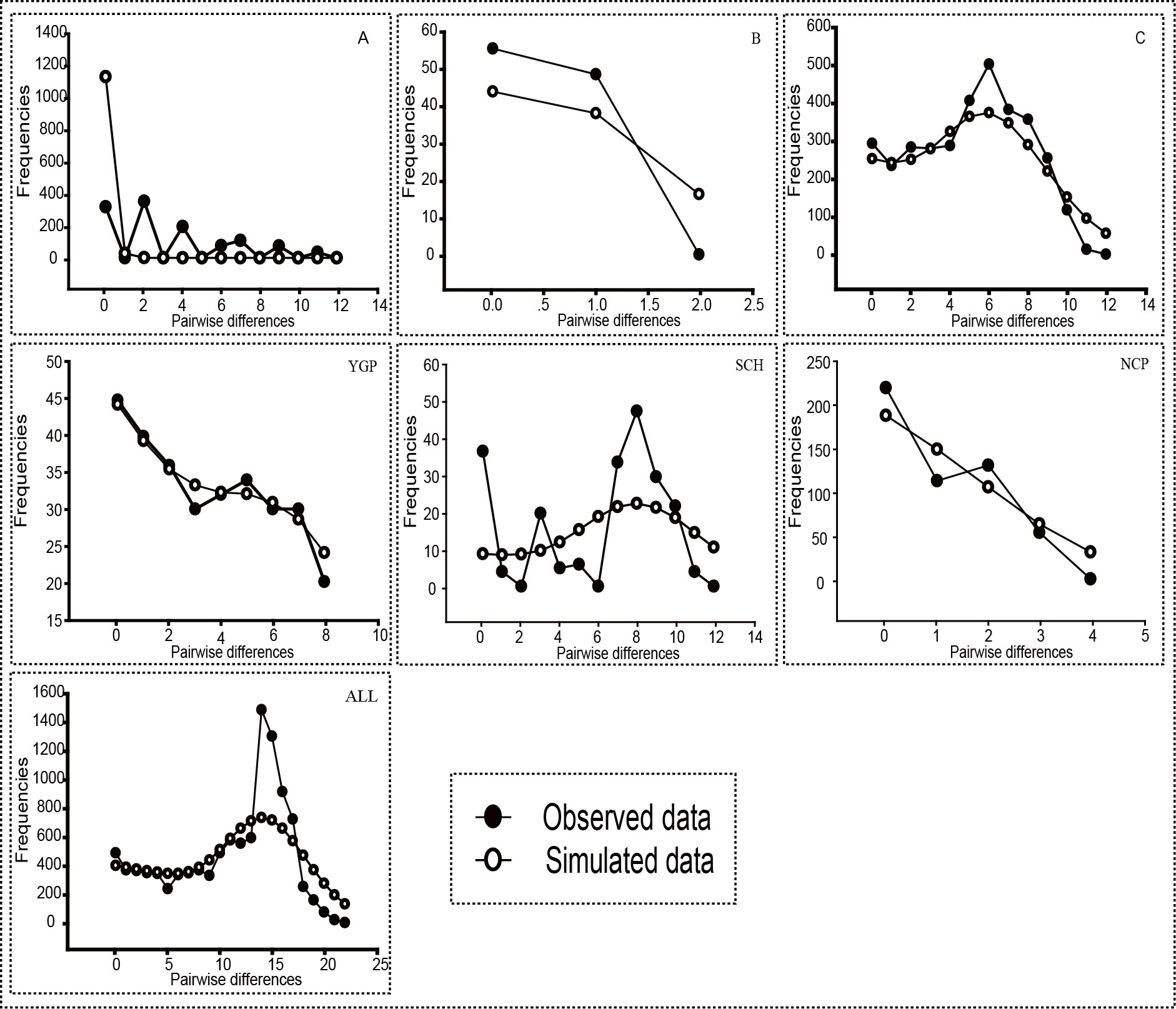

Supplement: Additional file 5 — Results of mismatch distributions for all populations, three lineages and three geographical units. The full circles show observed values whereas the open circle represents expected values. [file 1471-2148-13-241-S5.doc]
